# Acetylcholine release in mouse hippocampal CA1 preferentially activates inhibitory-selective interneurons via α4β2* nicotinic receptor activation

**DOI:** 10.3389/fncel.2015.00115

**Published:** 2015-04-13

**Authors:** L. Andrew Bell, Karen A. Bell, A. Rory McQuiston

**Affiliations:** Department of Anatomy and Neurobiology, Virginia Commonwealth UniversityRichmond, VA, USA

**Keywords:** optogenetics, nicotinic receptor, CA1, hippocampus, interneuron-selective interneuron, disinhibition

## Abstract

Acetylcholine (ACh) release onto nicotinic receptors directly activates subsets of inhibitory interneurons in hippocampal CA1. However, the specific interneurons activated and their effect on the hippocampal network is not completely understood. Therefore, we investigated subsets of hippocampal CA1 interneurons that respond to ACh release through the activation of nicotinic receptors and the potential downstream effects this may have on hippocampal CA1 network function. ACh was optogenetically released in mouse hippocampal slices by expressing the excitatory optogenetic protein oChIEF-tdTomato in medial septum/diagonal band of Broca cholinergic neurons using Cre recombinase-dependent adeno-associated viral mediated transfection. The actions of optogenetically released ACh were assessed on both pyramidal neurons and different interneuron subtypes via whole cell patch clamp methods. Vasoactive intestinal peptide (VIP)-expressing interneurons that selectively innervate other interneurons (VIP/IS) were excited by ACh through the activation of nicotinic receptors containing α4 and β2 subunits (α4β2*). ACh release onto VIP/IS was presynaptically inhibited by M2 muscarinic autoreceptors. ACh release produced spontaneous inhibitory postsynaptic current (sIPSC) barrages blocked by dihydro-β-erythroidine in interneurons but not pyramidal neurons. Optogenetic suppression of VIP interneurons did not inhibit these sIPSC barrages suggesting other interneuron-selective interneurons were also excited by α4β2* nicotinic receptor activation. In contrast, interneurons that innervate pyramidal neuron perisomatic regions were not activated by ACh release onto nicotinic receptors. Therefore, we propose ACh release in CA1 facilitates disinhibition through activation of α4β2* nicotinic receptors on interneuron-selective interneurons whereas interneurons that innervate pyramidal neurons are less affected by nicotinic receptor activation.

## Introduction

The formation of new memories in the hippocampus is influenced by nicotinic receptor function. This is exemplified by observations that the encoding of memories can be enhanced by the exogenous activation of nicotinic receptors (Davis and Gould, 2006; Levin et al., 2009) and memory performance can be impaired by the injection of α4β2* (nicotinic receptors that contain α4 and β2 subunits but may include other types of subunits) or α7 nicotinic receptor antagonists directly into the hippocampus (Levin, 2002). Furthermore, dysfunction of both α4 and β2 nicotinic subunits in the hippocampus have been correlated with memory impairment associated with addiction, neurodegenerative disease and aging. In regard to nicotine addiction, the β2 nicotinic subunit in the hippocampus has been linked to chronic nicotine withdrawal (Davis and Gould, 2009) and high affinity nicotinic receptors in the hippocampus, which are likely composed of α4β2* subunits (Nguyen et al., 2004), are upregulated in smokers (Perry et al., 1999). In terms of aging and neurodegenerative disease, a significant loss of α4 nicotinic receptor subunit expression has been observed in aging mice (Gahring et al., 2005) and an 80% decrease in α4 subunit expression has been reported in Alzheimer’s patients (Kellar et al., 1987). Furthermore, beta amyloid protein, a protein that has been associated with the etiology of Alzheimer’s disease, can inhibit α4β2* receptors at low concentrations (Wu et al., 2004). Therefore, it appears that α4β2* nicotinic receptor function in the hippocampus is necessary for normal memory formation and their dysfunction may contribute to memory impairment associated with aging and neurodegenerative disease.

In the mammalian central nervous system, 11 nicotinic receptor subunits have been identified, 9 of which have been found in the hippocampus (Sudweeks and Yakel, 2000). A significant portion of the influence that nicotinic receptor activation has on hippocampal network function is likely due to the expression of nicotinic receptors on inhibitory interneurons. In particular, α7 (Alkondon et al., 1997; Jones and Yakel, 1997; Frazier et al., 1998b; McQuiston and Madison, 1999), α4β2* (McQuiston and Madison, 1999; Sudweeks and Yakel, 2000) and α2 nicotinic receptors (McQuiston and Madison, 1999; Sudweeks and Yakel, 2000; Jia et al., 2009) have been shown to be functionally expressed on various subsets of hippocampal interneurons. Furthermore, electrically-evoked release of Acetylcholine (ACh) has been shown to result in α7 nicotinic receptor mediated excitatory postsynaptic responses in CA1 interneurons (Alkondon et al., 1998; Frazier et al., 1998a). Based on the identification of α7-mediated nicotinic synaptic events and the prevalence of functional α7 nicotinic responses in interneurons, α7 subunits had been thought to be the primary subunits mediating nicotinic receptor activation of hippocampal inhibitory interneurons. However, more recent studies that utilized optogenetics to release ACh from medial septum/diagonal band of Broca (MS/DBB) axon terminals in mouse hippocampal CA1 showed that nicotinic EPSPs in interneurons were almost exclusively mediated by α4β2* nicotinic receptors (Bell et al., 2011). These latter studies fit with behavioral studies that suggested that α4β2* nicotinic receptors may have a significant role in nicotine enhancement of memory processing (Davis and Gould, 2006).

Given the importance of α4β2* nicotinic receptors in normal and pathophysiological nervous system function, it is crucial to identify neuronal types endogenously activated by nicotinic receptors. Although it is known that α4β2* nicotinic receptors on hippocampal CA1 interneurons can be activated by ACh release from MS/DBB terminals (Bell et al., 2011), the identity of the specific subsets of interneurons displaying α4β2* nicotinic responses remains unknown. Therefore, using Cre-driver mice, fluorescent protein reporter mice, and optogenetics, we have investigated the subsets of hippocampal CA1 interneurons that respond with nicotinic responses following the release of ACh.

## Methods

### Animals

Vip^tm1(cre)Zjh^/J (VIP-Cre, JAX Stock No. 010908), B6;129P2-*Pvalb*^tm1(cre)Arbr^/J (PV-cre, Jax Stock No. 008069), B6;129S6-Chat^tm1(cre)Lowl^/J (Chat-Cre, JAX Stock No. 006410), B6.Cg-Gt(ROSA)26Sor^tm3(CAG-EYFP)Hze^/J (YFP, Jax Stock No. 007903), and B6;129*S-*Gt(ROSA)26Sor^tm35.1(CAG-aop3/GFP)Hze^/J (Arch-GFP, Jax Stock No. 012735) mice (Hippenmeyer et al., 2005; Madisen et al., 2010; Rossi et al., 2011; Taniguchi et al., 2011) used in these studies were housed in an animal care facility approved by the American Association for the Accreditation of Laboratory Animal Care (AAALAC). Animal experimental procedures followed a protocol approved by the Institutional Animal Care and Use Committee of Virginia Commonwealth University (AD20205). This protocol adhered to the ethical guidelines described in The Care and Use of Laboratory Animals 8th Edition (Garber et al., 2011). All efforts were made to minimize animal suffering and to reduce the number of animals used.

### Breeding Strategies and Chat Immunofluorescence

Two different breeding strategies were developed for these studies. Studies examining nicotinic responses in interneuron subtypes utilized a triple cross consisting of Chat-Cre × VIP-Cre × YFP-reporter (CVY). Studies that utilized archaerhodopsin to silence specific interneuron subtypes in CA1 used a triple cross consisting of Chat-Cre × VIP-Cre × Arch-GFP (CVA) (see Bell et al., 2015 for more details).

### Generation and Stereotaxic Injection of rAAV-Flex-rev-oChIEF-tdTomato into the MS/DBB of Chat-Cre Mice

A recombinant adeno-associated virus (rAAV, serotype 1, 1.05 or 1.8 × 10^13^ VC/ml titer) expressing FLEXed oChIEF-tdTomato was generated using a previously described method (Bell et al., 2011) in order to selectively express oChIEF-tdTomato in infected cells that also expressed Cre recombinase. Mice were initially anesthetized via intraperitoneal injection of ketamine (100 mg/kg IP) and xylazine (2.5 mg/kg IP). Anesthesia was maintained with O_2_ supplemented with isoflurane.

For injections into the MS/DBB, an incision was made in the skin along the midsagittal suture, and a small hole was drilled in the skull overlying the septum. An aluminosilicate glass pipette containing rAAV-Flex-rev-oChIEF-tdTomato was lowered to the level of the MS/DBB, 1.0 mm rostral to Bregma and infused at a rate of 100 nl/min using a software driven injectomate (Neurostar, Sindelfingen, Germany). In total, 7 × 100 nl injections were made between 3.75 and 5.0 mm in depth. For injections into the left nucleus basalis, coordinates were AP = −0.5, Lat = −1.6, Depth = 5.0–4.2 mm, and injection volume was 100 nl/site every 200 µm for a total of 500 nl. 10–15 days post viral injection, 42–70 day old mice were sacrificed for experimentation.

### Preparation of Hippocampal Slices

Brain slices were obtained by methods previously described (Bell et al., 2011). In brief, horizontal slices containing the mid-temporal hippocampus were cut at 350–450 µm on a Leica VT1200 (Leica Microsystems, Buffalo Grove, IL). Sections were incubated in a holding chamber kept at 36°C for 30 min and then allowed to return to room temperature. The holding and recording chamber solution consisted of normal saline (in mM): NaCl 125, KCl 3.0, CaCl_2_ 1.2, MgSO_4_ 1.2, NaHPO_4_ 1.2, NaHCO_3_ 25, glucose 25 bubbled with 95% O_2_/5% CO_2_. Recordings were performed at 32–35°C.

### Light-Evoked Release of ACh from MS/DBB Cholinergic Axon Terminals and Light-Evoked Silencing of CA1 Interneuron Subtype Populations

Cholinergic terminals expressing oChIEF-tdTomato were stimulated by blue light and interneurons expressing Arch-GFP were hyperpolarized by yellow light. Both light paths were transmitted through the epi-illumination light path of an Olympus BX51WI microscope and a 10× water immersion objective (0.3 NA). Blue light flashes (1 ms in duration) and yellow light pulses (4 s in duration) were generated from light-emitting diodes (LEDs) (UHP-microscope-LED-460 or UHP-T-LED-White filtered by an HQ 575/50x excitation filter, respectively, Prizmatix Modiin-Ilite, Givat Shmuel, Israel). Blue or yellow light exiting the LEDs were reflected or passed through a dichroic mirror (515dcxru, Chroma Technology, Bellows Falls, VT, USA) and were focused into the epi-illumination light path of the Olympus BX51WI microscope and back aperture of the 10x water immersion objective (0.3 NA) using an optiblock beam combiner (Prizmatix) and a dichroic mirror (700dcxxr, Chroma Technology, Bellows Falls, VT, USA) in the filter turret.

### Electrophysiological Measurements

Whole cell patch clamp recordings from hippocampal CA1 interneurons were performed using patch pipettes (2–5 MΩ) pulled from borosilicate glass (8250 1.65/1.0 mm) on a Narishige PC-10 pipette puller filled with (in mM): KCl 55, KGluc 70, NaCl 8, MgATP 2, NaGTP 0.1, HEPES 10, BAPTAK_4_ 2, QX314 chloride 10, biocytin 0.1% or KGluc 130, NaCl 8, MgATP 2, NaGTP 0.1, HEPES 10, BAPTAK_4_ 0.1, biocytin 0.1%. Elevated intracellular KCl, BAPTAK_4_, and the inclusion of QX314 in the intracellular solution were used for the measurement of spontaneous inhibitory postsynaptic currents (IPSCs), which appear as inward or negative going currents in voltage clamped current recordings (*V_h_* = −70 mV). Otherwise, a standard KGluc and lower BAPTAK_4_ intracellular solutions where used to measure membrane potential responses. Membrane potentials and/or currents were measured with a Model 2400 patch clamp amplifier (A-M Systems, Port Angeles, WA) and converted into a digital signal by a PCI-6040E A/D board (National instruments, Austin, TX). WCP Strathclyde Software was used to store and analyze membrane potential and current responses on a PC computer (courtesy of Dr. J Dempster, Strathclyde University, Glasgow, Scotland). To detect and analyze spontaneous inhibitory postsynaptic currents (sIPSCs), miniAnalysis (Synaptosoft, Fort Lee, NJ) was used. Further analysis was performed with Originpro 8.1 (OriginLab Corp., Northampton, MA, USA), Excel (Microsoft, Redmond, WA) and SPSS 20.0 (IBM, Armonk, NY).

### Immunofluorescence: Morphological Reconstruction of Interneurons Displaying Nicotinic and Muscarinic Responses and Amplification of Fluorescent Markers

Slices were fixed in 4% paraformaldehyde (Boston BioProducts) and incubated with streptavidin Alexa Fluor 633 (Life Technologies, Invitrogen) in phosphate buffered saline (PBS) with Triton-X 100 as previously described (Bell et al., 2011). Processed slices were then reconstructed using a Zeiss LSM 710 confocal microscope (Carl Zeiss, Jena, Germany). Alexa Fluor 633 was excited with the 633 nm line of a HeNe 5 mW laser and cells were visualized using a 20× dry lens (0.8 N.A., voxel dimensions 0.2 × 0.2 × 1.1 µm). The imaged interneurons were traced using the Autoneuron module within the Neurolucida program (MBP, Burlington, VT). For amplification of YFP-labeled interneurons, 1:200 dilution of rabbit anti-GFP conjugated to Alexa Fluor 488 (Life Technologies, Invitrogen) in goat blocking buffer (10% normal serum, 2% bovine serum albumin, 0.4% Triton-X 100 in 0.1 M phosphate buffer) was added to fixed and washed slices for overnight incubation. Before and after primary and secondary antibody incubations, slices were washed in PBS. Slices were mounted in either Prolong Gold® (Life Technologies, Invitrogen) or VECTASHIELD® hard mount (Vector Laboratories).

### Statistics and Data Analysis

Data were analyzed using WCP software and miniAnalysis for the electrophysiological measurements. Statistics were performed using SPSS 20.0 (IBM, Armonk, NY). Statistical significances for groups of 3 or more were determined using a one-way ANOVA with Bonferroni *post hoc* tests. The averaged statistical significances for groups of 2 were determined with two-tailed *t*-tests. For averaged time-dependent sIPSC frequency data, a one-way ANOVA was done to test whether the averaged sIPSC frequency changed over the course of each experiment. Differences were determined to be statistically significant for *p* values less than 0.05. All data was reported as the mean, standard error of the mean (SEM). Asterisks were as follows unless otherwise noted, ***p < 0.001, **p < 0.01, *p < 0.05.

### Chemicals

All chemicals were purchased from VWR unless otherwise indicated. VU 10010 (M_4_-selective positive allosteric modulator), SR 95531 hydrobromide (Gabazine, GABA_A_ antagonist), Baclofen (GABA_B_ antagonist), QX314 chloride (intracellular sodium channel blocker), and AF-DX 116 (selective M_2_- muscarinic receptor antagonist) were obtained from Tocris Bioscience (Ellisville, Missouri) and 6, 7-Dinitroquinoxaline-2, 3-dione (DNQX, AMPA receptor antagonist), DL-2-Amino-5-phosphono pentanoic acid (APV, NMDA receptor antagonist) from Ascent Scientific (Bristol, U.K.). Biocytin (B-1592) was purchased from Life Technologies (Invitrogen).

## Results

### ACh Released from MS/DBB Terminals Selectively Produced α4β2* Nicotinic Responses in VIP Interneuron-Selective Interneurons

**Figure 1:**
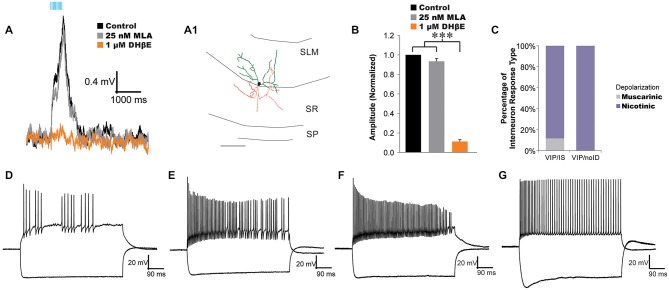
Acetylcholine (ACh) release activates α4β2*-nicotinic receptors on vasoactive-intestinal peptide (VIP) interneuron-selective interneurons. (A) VIP interneuron-selective (IS) interneuron (morphology**, A1**) responded to optogenetically released ACh with fast depolarizations (black traces, 10 × 20 Hz) that were inhibited by 1 µM DHβE (orange traces) but not 25 nM MLA (gray trace). **(B)** All VIP interneurons not displaying basket cell morphology were unaffected by bath application of 25 nM MLA (gray bar, *n* = 12) but were blocked by 1 µM DHβE (orange bar, one-way ANOVA, *p* < 0.001, Bonferroni *post hoc* *p* < 0.001, *n* = 20). **(C)** Histogram illustrating the distribution of response types across VIP expressing interneurons: VIP/IS cells, and VIP/noID. Most VIP/IS cells (19 of 21) and all non-visually identified VIP cells (19 of 19) exhibited nicotinic-dependent depolarizations (purple). **(D–F)** Example of VIP interneurons with nicotinic responses that displayed irregular action potential firing patterns that decreased in amplitude in response to depolarizing current injection. Negative current pulses produced hyperpolarizing membrane responses with little or no voltage sag. **(G)** A VIP interneuron that produced accommodating regular action potential firing patterns to depolarizing current injection and a hyperpolarizing sag in response to negative current injection.

There are two types interneurons that express vasoactive-intestinal peptide (VIP) in hippocampal CA1: those that exclusively innervate other interneurons (interneuron-selective interneurons, VIP/IS) and those that innervate the perisomatic region of pyramidal neurons (VIP basket cells, VIP/BC) (Acsády et al., 1996b). While nicotinic responses appear to occur in neocortical VIP interneurons (Arroyo et al., 2012), little is known about how hippocampal VIP interneurons respond to ACh released from MS/DBB cholinergic terminals. Therefore, we investigated the actions of ACh release on VIP interneurons using whole cell patch clamp recordings and optogenetics in acute mouse hippocampal brain slices. To target whole cell patch clamp recordings from VIP interneurons, we utilized CVY animals (see methods) that expressed YFP in VIP interneurons. To optogenetically release ACh from MS/DBB cholinergic terminals in hippocampal brain slices, we expressed the excitatory optogenetic protein oChIEF-tdTomato in MS/DBB cholinergic neurons through Cre-dependent AAV mediated transfection. Following whole cell patch clamp measurements, interneurons were morphologically reconstructed to determine if the interneuron from which we recorded was either a perisomatic projecting basket cell or an interneuron-selective interneuron (Figure 1A, VIP/IS). Cells with incomplete morphology were labeled “noID” (VIP/noID).

**Figure 2:**
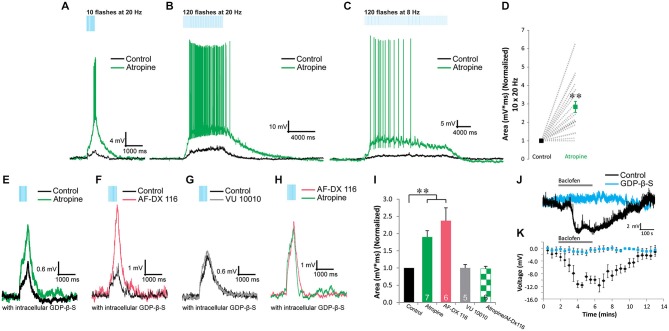
Presynaptic M_2_ autoreceptors inhibit the release of ACh onto α4β2* nicotinic receptors in VIP/IS interneurons. (A–C) Optogenetically released ACh produced depolarizing responses to short/fast (**A**, 10 × 20 Hz), prolonged/fast (**B**, 120 × 20 Hz), and prolonged/slow (**C**, 120 × 8 Hz) blue light flashes (black traces). The depolarizations were potentiated by 10 µM atropine (green traces). **(D)** Atropine (green) significantly potentiated the area of the nicotinic response (normalized to control, black, 10 × 20 Hz) (*t*-test, *p* < 0.001, *n* = 0). **(E)**. Inclusion of GDP-β-S (black trace) in the intracellular recording solution did not inhibit atropine (green trace) potentiation of the nicotinic responses. **(F)** M_2_ antagonist AF-DX 116 (500 nM, red trace) potentiated nicotinic responses when GDP-β-S was included in the intracellular solution. **(G)** M_4_ positive allosteric modulator VU 10010 did not affect nicotinic responses (one-way ANOVA, ns, *n* = 5). **(H)** M_2_ antagonist AF-DX 116 (500 nM, red trace) occluded atropine (green trace) potentiation of nicotinic responses. **(I)** Histogram showing that atropine (GDP-β-S) (one-way ANOVA, *p* < 0.001, Bonferroni *post hoc* test *p* < 0.001, *n* = 7) and AF-DX 116 (GDP-β-S) (Bonferroni *post hoc* test *p* < 0.001, *n* = 6) significantly potentiated nicotinic responses. Atropine (green checkers) did not significantly increase nicotinic responses previously potentiated by AF-DX 116 (GDP-β-S) (*t*-test, ns, *n* = 6). **(J)** Application of 10 µM baclofen hyperpolarized VIP interneurons (black trace) but not when GDP-β-S (blue trace) was included in the intracellular solution. **(K)** All VIP interneurons tested produced significant hyperpolarizations when exposed to baclofen (black dots, one-way ANOVA, *p* < 0.001, Bonferroni *post hoc* *p* < 0.01 for time points 4–7 min, *n* = 4). Recordings that included GDP-β-S in the patch pipette did not respond to baclofen application (blue dots, one-way ANOVA, ns, *n* = 4).

In a previous study, we demonstrated that all morphologically identified VIP basket cells responded to ACh release with a muscarinic receptor-dependent slow depolarization completely inhibited by atropine (Bell et al., 2015). In the present study, we attempted to preferentially record from VIP/IS cells by targeting recordings to small VIP fluorescent neurons in the middle of the stratum lacunosum-moleculare (SLM). These SLM interneurons had smaller cell bodies (≤10 um) that have been correlated with VIP/IS cells and not VIP basket cells (Acsády et al., 1996a). In most VIP/IS cells (19 of 21) and all small cell bodied SLM VIP/noID cells (15 of 15) optogenetically released ACh resulted in depolarizations that increased in amplitude in the presence of atropine (Figures 1C, 2A–E,I). The nicotinic responses were not significantly affected by bath application of the α7 nicotinic receptor antagonist MLA (25 nM, Figures 1A,B, *gray, n* = 12), but they were blocked by bath application of the α4β2* nicotinic receptor antagonist DHβE (1 µM, Figures 1A,B, *orange*, one-way ANOVA, *p* < 0.001, Bonferroni *post hoc* *p* < 0.001, *n* = 20) suggesting that they were mediated by α4β2* nicotinic receptors (Bell et al., 2011). Anatomical reconstruction of some nicotinic responding interneurons displayed morphology similar to that described for type 2 VIP/IS interneurons (Figure 1A; Acsády et al., 1996a) whereas others could not be definitively correlated with previously described classes of VIP/IS interneurons because of limited axonal morphology. Electrophysiological properties of nicotinic responding VIP interneurons were diverse. The average resting membrane potential for nicotinic responding VIP interneurons was −73.9 mV ± 0.8 mV (*n* = 30) and the average input resistance was 395.3 MΩ, ± 20.8 MΩ (*n* = 30). The nicotinic responsive interneurons showed no consistent active membrane properties. Some interneurons displayed irregular action potential firing patterns (Figures 1D–F) whereas others had regular accommodating action potential firing patterns (Figure 1G) (average accommodation ratio (last interval/first interval) 2.75 ± 0.84, *n* = 30). Many nicotinic receptor responding interneurons had decreasing action potential amplitudes during depolarizing test pulses (ratio last/first = 0.34, ± 0.07, *n* = 30) (Figures 1D,F). Furthermore, different nicotinic responding VIP interneurons could either display a voltage sag (Figure 1G) or it could be absent during hyperpolarizations (Figures 1D,F) (average sag ratio of 1.09 ± 0.01, *n* = 30). Therefore, nicotinic responding VIP interneurons could not be classified based on electrophysiological properties. In contrast to VIP/IS interneurons, endogenous nicotinic excitatory responses were not observed in VIP basket cells, parvalbumin interneurons, or pyramidal neurons (Bell et al., 2015, data not shown). Thus, nicotinic responses resulting from ACh release may preferentially occur in interneuron-selective interneurons.

### Nicotinic Responses in VIP/IS Interneurons are Presynaptically Inhibited by M_2_ Autoreceptors

In order determine whether muscarinic receptors contributed to depolarizing responses in VIP/IS interneurons, we bath applied atropine to inhibit muscarinic receptors. Rather than inhibiting the depolarization, atropine potentiated this response (Bell et al., 2011). This potentiation was independent of the number (10—Figure 2A vs. 120—Figures 2B,C) or frequency of pulses (20 Hz—Figures 2A,B vs. 8 Hz—Figure 2C). In some VIP/IS interneurons (5 of 20), previously subthreshold nicotinic responses could become suprathreshold following the application of atropine (Figures 2A–C). On average, the presence of atropine resulted in a 283 ± 30% increase in the area of the response (Figure 2D, *t*-test, *p* < 0.001, *n* = 20). To test whether this potentiation was due to a presynaptic or postsynaptic mechanism, we blocked postsynaptic G-protein coupled signaling by substituting GTP with GDP-β-S in the intracellular solution. When postsynaptic G-protein signaling was blocked, atropine continued to potentiate the nicotinic response (Figures 2E,I, one-way ANOVA, *p* < 0.001, Bonferroni *post hoc* test *p* < 0.001, *n* = 7) suggesting that ACh release from MS/DBB terminals onto nicotinic receptors is inhibited by presynaptic muscarinic autoreceptors.

We next determined whether GDP-β-S effectively blocked postsynaptic G-protein coupled signaling in our experimental system. To do this we bath applied the GABA_B_ agonist baclofen (10 µM) and measured hyperpolarizing responses in VIP/IS interneurons. In all cells where GTP was included in the intracellular solution (Figures 2J,K, *black*), baclofen caused a large sustained hyperpolarization (average amplitude at 4.5 min application = −11.6 ± 0.8 mV, one-way ANOVA, *p* < 0.001, Bonferroni *post hoc* *p* < 0.01 for time points 4–7 min, *n* = 4). In contrast, baclofen had no significant effect on the membrane potential of VIP/IS interneurons in which GDP-β-S was included in the intracellular solution (average amplitude at 4.5 min application = −1.1 ± 0.9 mV, Figures 2J,K, *blue*, one-way ANOVA, ns, *n* = 4). Therefore, inclusion of GDP-β-S in the intracellular solution was effective in blocking postsynaptic G-protein coupled signaling and the atropine mediated potentiation of the nicotinic response was at least in part mediated by a presynaptic mechanism.

To examine which muscarinic receptor subtype mediated this presynaptic inhibition, we tested whether the M_2_-selective antagonist AF-DX 116 or the M_4_ positive allosteric modulator VU 10010 affected the nicotinic response. M_4_ receptors appeared to have no role in mediating presynaptic inhibition as VU 10010 (5 µM) had no effect on nicotinic response amplitudes (Figures 2G,I one-way ANOVA, ns, *n* = 5). In contrast, AF-DX 116 (500 nM) (Figures 2F,I) significantly increased the size of nicotinic responses suggesting that ACh release from MS/DBB terminals onto VIP/IS interneurons was inhibited by presynaptic M_2_ receptors (Figure 2I, one-way ANOVA, *p* < 0.001, Bonferroni *post hoc* test *p* < 0.001, *n* = 6). The magnitude of the potentiation by AF-DX116 was similar to that produced by atropine (Figure 2I) suggesting M_2_ receptors were involved. Furthermore, prior application of AF-DX116 occluded the effect of atropine on nicotinic response amplitudes (Figures 2H,I, *t*-test, ns, *n* = 6). Therefore, M_2_ muscarinic receptors mediate most if not all of the inhibition of ACh release.

### Postsynaptic Targets of Interneurons Excited by Nicotinic Receptor Activation

**Figure 3:**
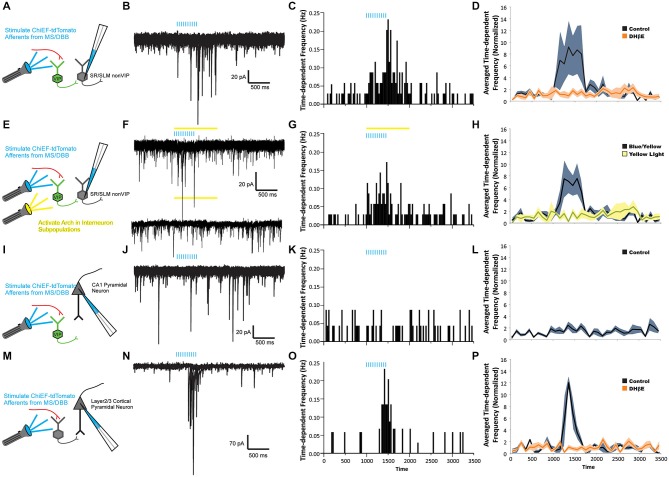
**ACh release drives nicotinic receptor-mediated feedforward inhibition onto CA1 interneurons but not CA1 pyramidal neurons. (A,E,I,M)** Schematic drawings of four recording paradigms. sIPSCs were recorded in response to ACh release in non-VIP CA1 interneurons located at the border of SR and SLM **(A–H)**, in CA1 pyramidal neurons **(I–L)**, or in layer 2/3 neocortical pyramidal neurons **(M–P)**. All recordings were performed in the presence of 10 µM atropine, 30 µM DNQX, and 50 µM APV. **(B)** Voltage clamp recordings (*V_h_* = −70 mV) demonstrating that optogenetic release of ACh (blue bars, 10 × 20 Hz) increased the number of sIPSCs observed in CA1 non-VIP interneurons. **(C)** A peristimulus time histogram (PSTH) illustrating the increase in time-dependent sIPSC frequency in nonVIP interneurons following ACh release. **(D)** An increase in the averaged time-dependent sIPSC frequency across all non-VIP interneurons (black line, gray shading = S.E.M, one-way ANOVA compared to baseline, *p* < 0.01, *n* = 23) was completely blocked by 1 µM DHβE (orange line, orange shading = SEM, one-way ANOVA, ns compared to baseline, *n* = 8). **(F)** Voltage clamp recordings showing that suppression of VIP interneurons by yellow light activation of Arch (yellow bar) did not inhibit the increase in sIPSC frequency following ACh release (blue bars). **(G)** PSTH showing time-dependent sIPSC frequency following ACh release persisted in the presence of yellow light (yellow bar). **(H)** The increased averaged time-dependent sIPSC frequency measured across all non-VIP interneurons (black line, gray shading = S.E.M, one-way ANOVA, *p* < 0.001, *n* = 21) was not suppressed by activation of Arch in VIP interneurons. **(J)** Voltage clamp recordings from a CA1 pyramidal neuron showed no change in sIPSC frequency following ACh release (blue bars). **(K)** PSTH demonstrated that the time-dependent sIPSC frequency was unchanged following ACh release in an individual CA1 pyramidal neuron. **(L)** Averaged time-dependent sIPSC frequency across all CA1 pyramidal cell recordings demonstrated no change sIPSC frequency following ACh release (black line, gray shading = S.E.M, one-way ANOVA, ns, *n* = 42). **(N)** Voltage clamp recordings from a neocortical pyramidal neuron demonstrated that ACh release produced an increase in sIPSC frequency. **(O)** PSTH demonstrated that the time-dependent frequency of sIPSCs in the neocortical pyramidal neuron increased following ACh release. **(P)** The averaged time-dependent sIPSC frequency in all measured neocortical pyramidal neurons increased following ACh release (black line, gray shading = S.E.M., one-way ANOVA, *p* < 0.001, *n* = 5) and was blocked by 1 µM DHβE (orange line, orange shading = S.E.M., one-way ANOVA, ns, *n* = 5).

We next investigated the downstream effects of the interneurons that were excited by nicotinic receptor activation. We recorded changes produced by nicotinic receptor activation on the frequency of sIPSCs in different CA1 interneuron subtypes located in the dendritic layers of CA1 (Figures 3A–H), CA1 pyramidal cells (Figures 3I–L), and layer 2/3 neocortical pyramidal neurons (Figures 3M–P). To increase the amplitudes of sIPSCs and improve their detection, recordings were made with elevated intracellular chloride, QX314 to block action potentials that escaped voltage clamp, and elevated BAPTA to maintain low intracellular calcium levels. Under these recording conditions, voltage clamped sIPSCs resulted in inward currents. In all experiments, muscarinic receptors were blocked by atropine, ionotropic glutamate receptors were blocked by DNQX (30 µM) and APV (50 µM), and GABA_B_ receptors were blocked by CGP54626 (1 µM). ACh was released by 10 × 1 ms flashes of blue light at 20 Hz at 1 min intervals. Peristimulus-time histograms for sIPSCs were constructed to detect any changes in the average time-dependent sIPSC frequency.

Following the activation of nicotinic receptors by the optogenetic release of ACh, approximately half of dendritically located hippocampal CA1 interneurons (23 of 49 non VIP expressing, morphologically unidentified) displayed an increase in average sIPSC frequency (Figures 3B–D). One example illustrated that ACh release caused the time-dependent sIPSC frequency to increase from 0.035–0.231 Hz (Figure 3C). When normalized and averaged across all interneuron recordings, the average time-dependent sIPSC frequency increased by 574% (± 283%) during and immediately after 500 (ms after last flash) light flashes (Figure 3D, one-way ANOVA, *p* < 0.01, *n* = 23). This increase in frequency was subsequently blocked by bath application of DHβE (1 µM) suggesting that α4β2* nicotinic receptors mediated the excitation of presynaptic interneurons (Figure 3D, one-way ANOVA, ns relative to control, *n* = 8). In order to determine whether VIP interneurons were the exclusive presynaptic interneurons activated by ACh release, Arch-GFP was expressed in VIP interneurons (Bell et al., 2015). When Arch was expressed in VIP-Cre interneurons, we found that a yellow light pulse did not affect the increased frequency of sIPSCs produced by ACh release (Figures 3F–H, one-way ANOVA, ns, *n* = 21). This suggested that other non-VIP/IS interneurons also respond to ACh release through the activation of α4β2* nicotinic receptors.

Hippocampal CA1 interneurons are inhibited by interneuron-selective interneurons as well as interneurons that innervate both interneurons and pyramidal cells. Therefore, we examined whether the non-VIP interneurons activated by nicotinic receptors inhibited pyramidal neurons in addition to interneurons. In all morphologically identified CA1 pyramidal neurons examined (*n* = 42), no change in time-dependent sIPSC frequency was observed during or following the release of ACh (Figures 3J–L). Furthermore, normalization and averaging the time-dependent sIPSC frequency across all pyramidal neurons showed no significant increase in sIPSC frequency following ACh release compared to baseline sIPSC frequency (Figure 3L, one-way ANOVA, ns, *n* = 42). These observations were somewhat unexpected as previous studies had suggested that ACh release may activate nicotinic receptors on interneurons that innervate CA1 pyramidal neurons (Nagode et al., 2011). Furthermore, neocortical interneurons that innervate layer 2/3 pyramidal neurons have been shown to be excited by ACh release onto nicotinic receptors (Arroyo et al., 2012). Therefore, to test whether in our system we can activate nicotinic receptors on interneurons that innervate pyramidal neurons, we expressed oChIEF-tdTomato in cholinergic neurons of the nucleus basalis. This permitted us to record from layer 2/3 pyramidal neurons of the neocortex and measure changes in sIPSC frequency following ACh release (Figures 3N–P). Similar to previous reports (Arroyo et al., 2012), ACh release increased the time-dependent frequency of sIPSC in neocortical pyramidal neurons (Figures 3N–P, one-way ANOVA, *p* < 0.001, *n* = 5) and the increased frequency was blocked by DHβE (1 µM, one-way ANOVA, ns relative to control, *n* = 5). Therefore, our data suggested that ACh release onto nicotinic receptors in hippocampal CA1 preferentially activates interneurons that innervate other interneurons rather than interneurons that inhibit pyramidal neurons.

## Discussion

Our data suggest that nicotinic transmission preferentially engages specific networks in hippocampal CA1. Without affecting pyramidal neurons, nicotinic cholinergic transmission activated very specific groups of interneurons in hippocampal CA1 that selectively inhibit other interneurons (interneuron-selective interneurons). Release of ACh appeared to activate nicotinic receptors on both VIP and non-VIP interneuron-selective interneurons. Importantly, nicotinic responses were potently controlled by M2 muscarinic receptor-driven presynaptic inhibition.

Our data confirmed previous reports from our laboratory that suggested that hippocampal CA1 interneurons can be excited by ACh released onto postsynaptic α4β2* nicotinic receptors (Bell et al., 2011). Our current studies extend these findings by demonstrating that α4β2*-mediated nicotinic responses preferentially occurred in morphologically identified VIP/IS interneurons. We did not observe nicotinic responses in VIP/BCs, PV interneurons or pyramidal neurons. Furthermore, disynaptic IPSCs produced by nicotinic receptor activation were only detected in interneurons and not pyramidal cells. Importantly, these disynaptic IPSCs were not inhibited by optogenetic silencing of VIP interneurons suggesting that other non-VIP-expressing interneuron-selective interneurons were also excited by the release of ACh onto α4β2* nicotinic receptors (Acsády et al., 1996b; Gulyás et al., 1996). This may be expected as VIP/IS interneurons only make up a minority (37%) of interneuron-selective interneurons (Acsády et al., 1996a). Importantly, a significant portion of the interneurons innervated by interneuron-selective interneurons target the dendrites of CA1 pyramidal neurons (Acsády et al., 1996a; Gulyás et al., 1996). Therefore, nicotinic disinhibition in hippocampal CA1 may facilitate dendritic integration of synaptic inputs in CA1 pyramidal neurons.

It should be noted that the triple crosses in the experiments described above should result in the expression of Arch-GFP in both VIP and cholinergic neurons (CVA animals). This could make interpretation of the results from these experiments difficult as an inhibition of the nicotinic receptor-driven response might result from the inhibition of VIP neurons or the inhibition of cholinergic terminals. However, activation of Arch in our preparation had no effect on the nicotinic driven inhibitory postsynaptic currents measured in interneurons. Thus, Arch must not be expressed at sufficient concentrations in cholinergic terminals to influence optogenetically-driven release of ACh.

Our nicotinic data were inconsistent with previous findings that suggested that CA1 pyramidal neurons (~20%) (Gu and Yakel, 2011) and interneurons that innervate pyramidal neurons (Nagode et al., 2011) may be excited by ACh released onto nicotinic receptors. Although we have never observed a nicotinic response in recordings from 132 pyramidal neurons or 104 non IS interneuron subtypes, it is possible that we did not sample enough neurons or had a bias toward recording from particular interneuron subtypes. Furthermore, different injection methods and excitatory optogenetic proteins used between the different studies may have limited our ability to record nicotinic responses in pyramidal neurons or interneurons that innervated pyramidal cells. Alternatively, the studies that reported nicotinic responses in other cell types did not confirm the recorded neurons identity with *post hoc* anatomical reconstructions or electrophysiological characterization. Therefore, it remains possible that the nicotinic responses (Gu and Yakel, 2011) and nicotinic receptor-driven disynaptic IPSCs (Nagode et al., 2011) measured in putative pyramidal neurons were actually recordings made from interneurons and not pyramidal cells. Another possibility for the lack of nicotinic receptor-driven disynaptic IPSCs in pyramidal neurons observed in our study may be that nicotinic responses in interneurons that target pyramidal neurons are mostly subthreshold. Regardless of these inconsistencies, our data suggest that a primary effect of α4β2* nicotinic receptor-mediated transmission is to excite interneuron-selective interneurons and aid in disinhibition of the hippocampal CA1 network.

Nicotinic receptor-mediated transmission in hippocampal CA1 has been shown to be potently inhibited by the release of ACh onto muscarinic receptors (Bell et al., 2011). Here we demonstrated that the muscarinic receptor inhibition of nicotinic transmission persisted when postsynaptic G-protein functioning was blocked—suggesting a presynaptic mechanism. This presynaptic inhibition of ACh release appears to be mediated by M2 muscarinic receptors, similar to findings in the thalamus (Sun et al., 2013). Importantly, muscarinic receptor presynaptic inhibition was potent and often prevented nicotinic responses from eliciting action potentials in interneuron-selective interneurons. Thus, therapeutic pharmacological blockade of M2 muscarinic receptors would be expected to facilitate nicotinic receptor-mediated influence on disinhibitory circuitry in hippocampal CA1.

Similar to our findings in the hippocampus, nicotinic receptor-mediated disinhibition has been observed in the auditory cortex (Letzkus et al., 2011). Some of the neocortical interneurons excited by ACh release expressed Chat, which has been shown to be expressed exclusively in VIP-expressing interneurons (Porter et al., 1998; Gonchar et al., 2008). However, unlike our studies, nicotinic transmission was observed in interneurons that innervated both pyramidal neurons and other interneurons (Arroyo et al., 2012). Therefore, the effect of nicotinic receptor-mediated transmission in the neocortex will likely be more complex than a primarily disinhibitory role that we hypothesize for the hippocampus.

### Summary

Optogenetic techniques have recently permitted more extensive studies on the effect of ACh release in various regions of the CNS. In particular, nicotinic responses to ACh release have been studied in several brain regions including the interpeduncular nucleus (Ren et al., 2011), hippocampus (Bell et al., 2011), striatum (English et al., 2011), neocortex (Letzkus et al., 2011; Arroyo et al., 2012), spinal cord (Lamotte d’Incamps et al., 2012) and thalamus (Sun et al., 2013). A common feature of these responses is that they activate GABAergic neurons. In some areas, nicotinic receptors activate GABAergic projection neurons (Ren et al., 2011; Sun et al., 2013). In other regions, they activate select types of inhibitory interneurons (Bell et al., 2011; English et al., 2011; Arroyo et al., 2012). The impact that physiologically activated nicotinic receptors have in each brain region will depend on the specific types of GABAergic neurons activated by ACh release. In the case of hippocampal CA1, interneuron-selective interneurons appear to be preferentially activated by nicotinic receptors suggesting a feedforward disinhibitory role for nicotinic receptor-mediated transmission.

## Conflict of Interest Statement

The authors declare that the research was conducted in the absence of any commercial or financial relationships that could be construed as a potential conflict of interest.
